# Bioinformatics analysis on multiple Gene Expression Omnibus datasets of the hepatitis B virus infection and its response to the interferon-alpha therapy

**DOI:** 10.1186/s12879-019-4720-x

**Published:** 2020-01-29

**Authors:** Zebin Zhu, Shanzhou Huang, Yixi Zhang, Chengjun Sun, Yunhua Tang, Qiang Zhao, Qi Zhou, Weiqiang Ju, Xiaoshun He

**Affiliations:** 1 Organ Transplant Center, The First Affiliated Hospital of USTC, Division of Life Sciences and Medicine, University of Science and Technology of China, Hefei, 230001 Anhui China; 2 Organ Transplant Center, The First Affiliated Hospital, Sun Yat-sen University, No. 58 Zhongshan Er Road, Guangzhou, 510080 Guangdong China; 3 Department of General Surgery, Guangdong General Hospital, Guangdong Academy of Medical Sciences, Guangzhou, 510030 Guangdong China; 4 Guangdong Provincial Key Laboratory of Organ Donation and Transplant Immunology, Guangzhou, 510080 Guangdong China; 5 Guangdong Provincial International Cooperation Base of Science and Technology (Organ Transplantation), Guangzhou, 510080 Guangdong China; 6 Department of General Surgery, Hui Ya Hospital of The First Affiliated Hospital, Sun Yat-sen University, Huizhou, 516081 Guangdong China; 7 Department of Liver Surgery, The First Affiliated Hospital, Sun Yat-Sen University, Guangzhou, 510080 Guangdong China

**Keywords:** Hepatitis B virus, Bioinformatics analysis, Differentially expressed gene

## Abstract

**Background:**

Hepatitis B virus (HBV) infection is a global health problem and interferon-alpha (IFN-α) is one of the effective therapies. However, little is known about the genetic background of the HBV infection or the genetic determinants of the IFN-α treatment response. Thus, we aim to explore the possible molecular mechanisms of HBV infection and its response to the IFN-α therapy with a comprehensive bioinformatics analysis.

**Methods:**

The Gene Expression Omnibus datasets (GSE83148, GSE84044 and GSE66698) were collected and the differentially expressed genes (DEGs), key biological processes and intersecting pathways were analyzed. The expression of the co-expressed DEGs in the clinical samples was verified by quantitative real time polymerase chain reaction (qRT-PCR).

**Results:**

Analysis of all the 3 datasets revealed that there were eight up-regulated and one down-regulated co-expressed DEGs following the HBV infection and after IFN-α treatment. In clinical samples, the mRNA level of HKDC1, EPCAM, GSN, ZWINT and PLD3 were significantly increased, while, the mRNA level of PLEKHA2 was significantly decreased in HBV infected liver tissues compared to normal liver tissues. PI3K-Akt signaling pathway, focal adhesion, HTLV-I infection, cytokine-cytokine receptor interaction, metabolic pathways, NF-κB signaling pathway were important pathways associated with the HBV infection and the response of IFN-α treatment.

**Conclusions:**

The co-expressed genes, common biological processes and intersecting pathways identified in the study might play an important role in HBV infection and response of IFN-α treatment. The dysregulated genes may act as novel biomarkers and therapeutic targets for HBV.

## Background

Hepatitis B virus (HBV) infection is one of the most common and serious infectious diseases in the world [1]. Chronic HBV has characteristics of high morbidity and are hard-to-heal; HBV-related hepatitis has lots of adverse impact on the patients’ quality of life, even causing death of the patients. At present, more than 240 million individuals, worldwide, are infected with chronic HBV. Amongst the untreated individuals with chronic HBV infection, 15 to 40% progress to cirrhosis, which may lead to liver failure or hepatic carcinoma [2]. The more serious problem is that 25% of individuals who acquire HBV as children develop primary cirrhosis or hepatic carcinoma as adults [3]. As HBV infection is ranked as the top health priorities of the world, the world health organization (WHO) has included viral hepatitis as its major public health priorities [4].

Chronic HBV infection is known to cause an immune-mediated liver damage which progresses to cirrhosis and hepatocellular carcinoma (HCC). Although vaccine and antiviral therapies have been used for some decades, the HBV infections were not thoroughly cured. Thus, it is crucial to study more about the underlying biological mechanisms of the progression of HBV infection. Interferon-alpha (IFN-α) has been the first-line of treatment for chronic HBV infection for decades due its low rates of drug resistance and high rates of HBsAg sero-clearance. However, only 30–40% of the chronic HBV patients benefits from the IFN-α therapy [5]. Studies have shown that genetic variations of the host may provide new approaches to predict responses to the IFN-α based therapy [6]. Therefore, it is critical to discover predictors for the outcomes of IFN-α treatment to improve the personalized therapy for the chronic HBV patients.

Gene profiling analyses have been used as a common method to identify the molecular mechanisms underlying the progression of diseases. Several studies [7–9] have used the gene array technologies to report different identifying factors involved in the HBV-related hepatic diseases. However, there are only a few studies about the relationship of HBV infection with gene expression profiling. In an attempt to explore the molecular mechanisms of HBV infection and discover predictors for outcomes of IFN-α treatment, we carried out a comprehensive bioinformatics analysis of the HBV infected and normal liver tissues, responders before and after the IFN-α treatment on the Gene-Cloud of Biotechnology Information (GCBI) bioinformatics platform. Subsequently, based on the comprehensive bioinformatics analysis, we determined several key differentially expressed genes (DEGs), biological processes and pathways that are closely associated with the HBV infection and response to the IFN-α treatment. Moreover, to validate the reliability of the microarray data, the expression of the DEGs was assessed by quantitative real time polymerase chain reaction (qRT-PCR) in the clinical samples.

## Methods

### Gene Expression Omnibus (GEO) datasets

GEO (https://www.ncbi.nlm.nih.gov/gds) is a public repository at the National Center of Biotechnology Information (NCBI) for storing data generated from high-throughput microarray experiments. We selected potential GEO datasets according to the following inclusion criteria: 1) specimens with histological diagnosis; 2) human liver tissues diagnosed as HBV-positive were used as the experimental group; 3) normal liver tissues (HBV-negative) used as controls; and 4) supported by GCBI analysis laboratory. And the exclusion criteria as follows: 1) data generated from other organisms; 2) expression profiling by qRT-PCR (or genome variation profiling by SNP array/SNP genotyping by SNP array); 3) analyses on platforms other than GPL570 or 4) sample size < 10.

We used the search terms “HBV infection” [MeSH Terms] and “*Homo sapiens*” [Organism] and “CEL” [Supplementary Files] and “Expression profiling by array” [DataSet Type] in the GEO datasets to determine potential datasets. Then, all the searched datasets were screened carefully according to the above inclusion and exclusion criteria. Finally, 3 GEO datasets, GSE83148, GSE84044 and GSE66698 were included in the present study.

### GCBI

GCBI (Shanghai, China, https://www.gcbi.com.cn) is a powerful platform that provides comprehensive bioinformatics analysis, and it can create a “gene knowledge base,” which involves GEO datasets. GCBI platform can systematically analyze GEO dataset-derived gene expression information, including more than 120 million copies of genomic samples [10]. In our study, GCBI was used to identify DEGs between the HBV infected and normal liver tissues, liver biopsy samples of IFN-α responders before and after treatment. In the DEG analysis module provided by the GCBI platform, DEGs with a fold expression change > 2 at cut-off values Q < 0.05 and *P* < 0.05 were identified. Venn diagrams were used to compare the top 50 DEGs from 3 cohorts by Venny (http://bioinfogp.cnb.csic.es/tools/venny/index.html). Based on the DEGs, biological functions were studied by means of gene ontology (GO) analysis and biochemistry pathways were evaluated by Kyoto Encyclopedia of Genes and Genomes (KEGG) analysis. The top 20 biological functions and signaling pathways are presented. Additionally, the core networks and pathway connections were identified using a pathway relation network module. Therefore, to determine the core genes in the networks, we utilized the Gene Co-expression Network module on the GCBI platform to construct gene co-expression networks for the DEGs. The significant GO biological process terms and KEGG pathways for the identified DEGs were evaluated using the Database for Annotation, Visualization, and Integrated Discovery (DAVID) and the WEB-based GEne SeT AnaLysis Toolkit (WebGestalt). For GO, KEGG and co-expression network analysis, the *P* < 0.05 threshold was used to determine statistical significance.

### Gene signal network analysis

Many studies have shown that the expression of genes is affected by one another. This interactive and mutually restrictive relationship constitutes a complex network of gene expression and regulation. Therefore, a gene’s upstream or downstream genes could be obtained by a gene signal network analysis of the entire KEGG pathway database. In the present study, we performed gene signal network analysis (GCBI Analyzing Lab) by using the GCBI platform (https://www.gcbi.com.cn/gclab/html/index) to determine hub genes. For gene signal network analysis, the *P* < 0.05 threshold was used to determine statistical significance.

### Tissue specimens, RNA extraction and qRT-PCR analysis

Ten normal liver tissues from the HBV-negative donor livers and 15 HBV-positive liver tissues from patients with HBV related hepatic cirrhosis were enrolled in our study to validate the expression levels of co-expressed DEGs.

Additional file 1: Table S1 shows the clinical characteristic of the 10 donors and 15 patients who underwent liver transplantation in detail. Prior patient consent and ethical approval from the ethics committee of The First Affiliated Hospital of Sun Yat-sen University were obtained. In accordance with the ethics guidelines and regulations, all methods were performed. We validated the 8 up-regulated co-expressed DEGs, including RRM2, HKDC1, EPCAM, GSN, CXCR4, MTHFD2, ZWINT, PLD3 and 1 down-regulated co-expressed DEG (PLEKHA2). HBV infection was diagnosed based on the patient’s laboratory testing results as being “HBsAg positive”, “HBV DNA positive” or “both positive”. Total RNA from the liver tissue specimens was extracted using the TRIzol reagent (Invitrogen, Carlsbad, California, USA), and qRT-PCR was performed with the SYBR® Green dye (TaKaRa, Shiga, Japan), following the manufacturer’s instructions. The primer sequences are provided in Additional file 2: Table S2. β-tubulin was used as a reference gene.

### Statistical analysis

Data are presented as the mean ± standard deviation for continuous variables. Analysis of variance and Student’s *t*-test were used to compare the differences between the groups. Analyses were carried out by the Statistical Package for the Social Science (SPSS) for Windows, version 22.0 (IBM, USA). Results were considered significant when a *P* value was less than 0.05 and all *P* values were two-sided.

## Results

### Study design

**Fig. 1:**
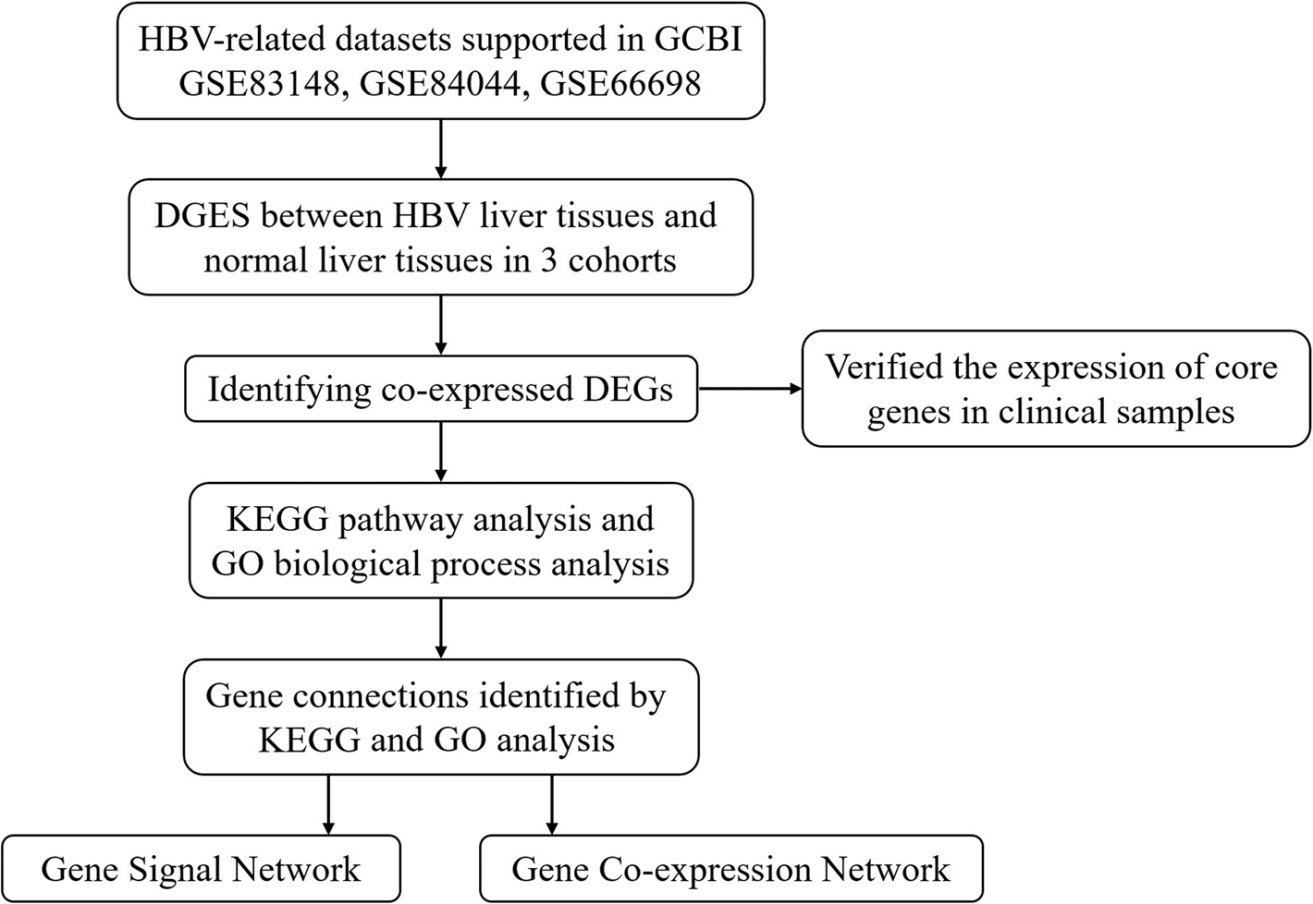
Flow chart of the study design

The flow chart of our study design is shown in Fig. 1. Our initial aim was to identify the core genes involved in the development of HBV infection. Using the 3 GEO datasets (GSE83148, GSE84044 and GSE66698) in the GCBI bioinformatics analysis platform, we extracted the gene expression data of the HBV infected and normal liver tissues to identify the DEGs. The co-expressed DEGs were identified from these 3 cohorts. Then, we verified the mRNA expression of the co-expressed DEGs in the clinical samples to confirm the results of the microarray analysis. The biological function and KEGG pathway analyses were performed on the 3 datasets. Moreover, we performed the gene signal network and gene co-expression network analyses to identify the gene connections between the different genes.

### Basic characteristics of samples in 3 datasets

GEO datasets GSE83148 (Cohort 1), GSE84044 (Cohort 2) and GSE66698 (Cohort 3) were used for the bioinformatics analysis of our study. Cohort 1 [11] contained 122 HBV infected and 6 normal liver tissues; the HBV infected liver samples were validated by positive HBsAg or serum HBV-DNA. Cohort 2 [12] contained 124 HBV infected and 6 normal liver tissues. All patients were diagnosed on the basis of the criteria recommended by the Asian Pacific Association for the Study of the Liver [13]. Cohort 3 [14] included 7 paired liver biopsy samples of the IFN-α responders before/after treatment and 3 other pre-treatment samples. All the 3 datasets were available in the GCBI bioinformatics analysis platform.

### DEGs of the HBV infected and normal liver tissues, liver biopsy samples of the IFN-α responders before and after treatment

**Fig. 2:**
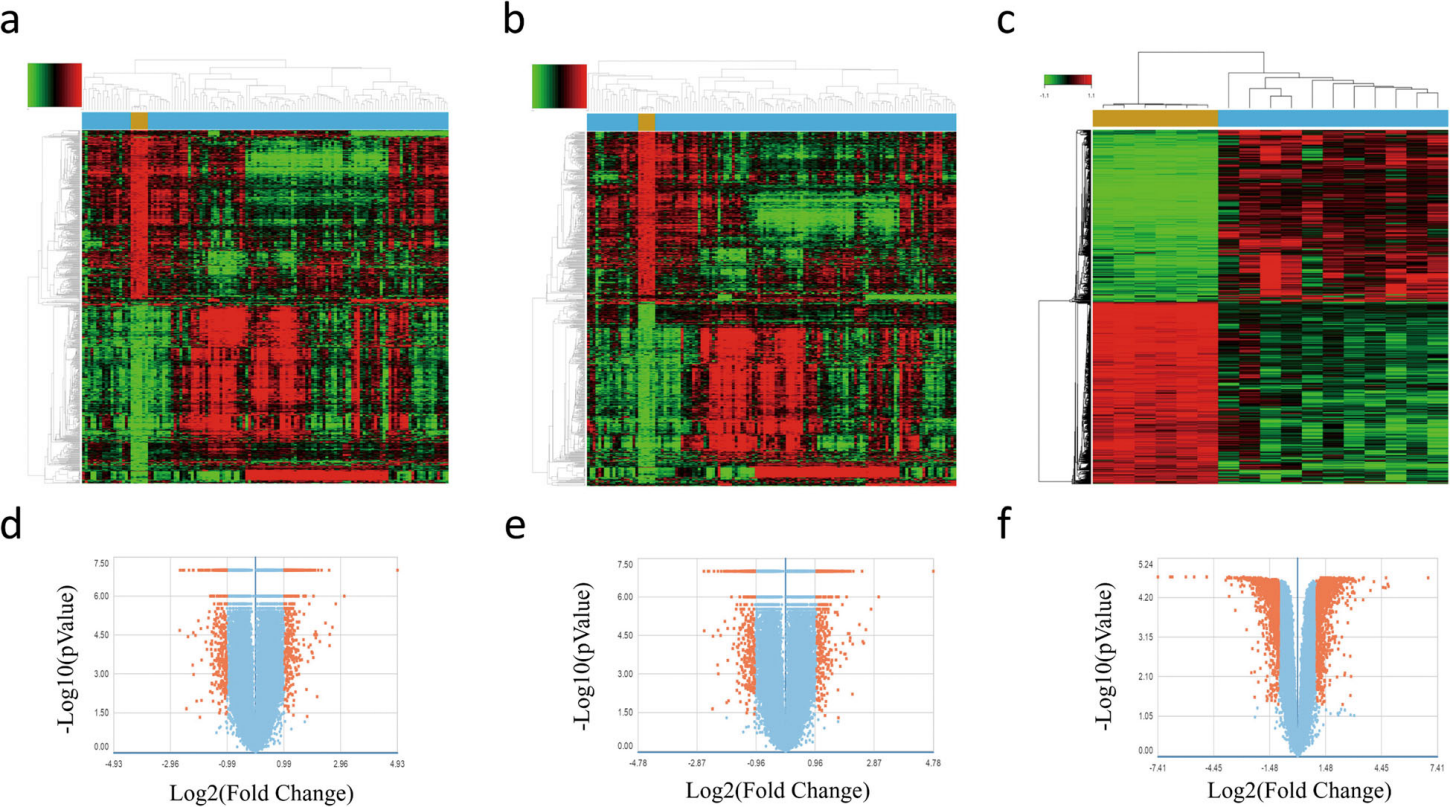
DEGs in HBV infected liver and liver biopsy samples of the IFN-α responders after treatment. **a**-**c** Heat maps for the potential DEGs between the HBV infected and normal liver tissues in the 3 cohorts. Heat maps for the potential DEGs in GSE83148 (containing 122 HBV infected and 6 normal liver tissues) (**a**), GSE84044 (containing 124 HBV infected and 6 normal liver tissues) (**b**) and GSE66698 (containing 7 paired liver biopsy samples of the IFN-α responders before/after treatment and 3 other pre-treatment samples) (**c**). **d**-**f** Volcano plot shows the DEGs between HBV infected and normal liver tissues in 3 cohorts (Fold change > 2, *P* < 0.05). Volcano plots for the potential DEGs in GSE83148 (**a**), GSE84044 (**b**) and GSE66698 (**c**)

**Fig. 3:**
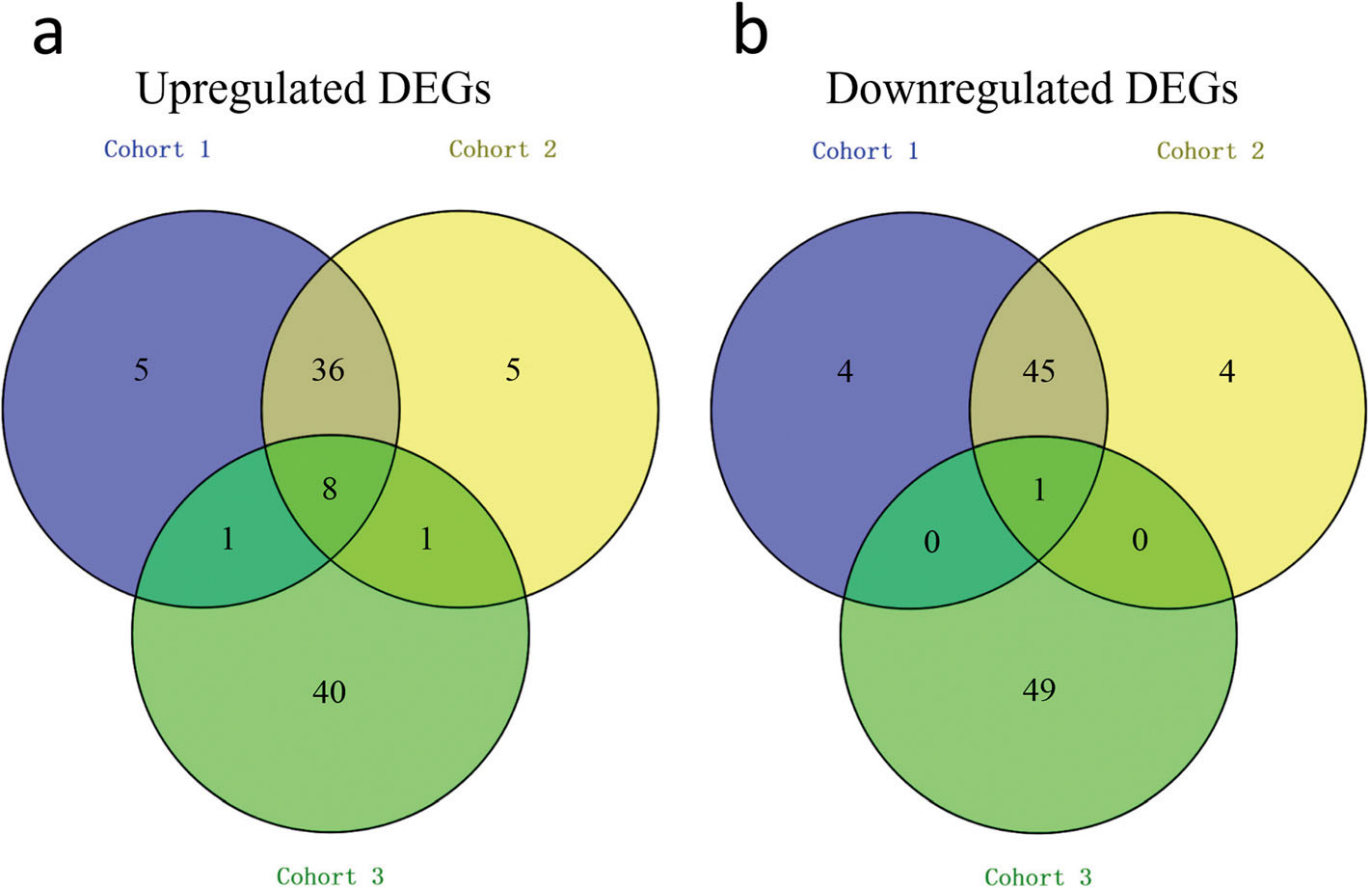
The Venn diagrams show the DEGs and the co-expressed genes among the 3 cohorts. The Venn diagram shows the top 50 up-regulated DEGs and co-expressed genes amongst cohort 1 (GSE83148), cohort 2 (GSE84044) and cohort 3 (GSE66698) (**a**). The Venn diagram shows the top 50 down-regulated DEGs and co-expressed genes amongst the 3 cohorts (**b**)

We identified 775, 742, and 2200 potential DEGs in GSE83148, GSE84044 and GSE66698 datasets, respectively (Fig. 2a-c). Table 1 shows the top 10 DEGs of the 3 cohorts. After removing the duplicate genes and expression values lacking specific gene symbols, we used the top 50 DEGs from GSE83148, GSE84044 and GSE66698 datasets to create a Venn diagram. The intersection of these 3 datasets in Fig. 3 shows that RRM2, HKDC1, EPCAM, GSN, CXCR4, MTHFD2, ZWINT, PPLD3 were the up-regulated co-expressed DEGs, and PLEKHA2 was the down-regulated co-expressed DEG in all the 3 cohorts. Every 2 datasets were checked for intersection for common genes, viz., SOX4 was co-expressed in Cohort 1 and Cohort 3; and the CBS gene co-expressed in Cohort 2 and Cohort 3; while, 36 other genes co-expressed in Cohort 1 and Cohort 2.

**Table 1:** Top 10 differentially expressed genes in 3 cohorts

Rank	Probe set ID	Gene symbol	Gene description	Regulation
Cohort 1				
1	224588_at	XIST	X inactive specific transcript	up
2	239380_at	C5orf27	chromosome 5 open reading frame 27	down
3	203032_s_at	FH	fumarate hydratase	down
4	238013_at	PLEKHA2	pleckstrin homology domain containing, family A, member 2	down
5	201909_at	RPS4Y1	ribosomal protein S4, Y-linked 1	down
6	209773_s_at	RRM2	ribonucleotide reductase M2	up
7	220116_at	KCNN2	small conductance calcium-activated channel subfamily N member 2	down
8	227614_at	HKDC1	hexokinase domain containing 1	up
9	1555175_at	PBLD	phenazine biosynthesis-like protein domain containing	down
10	204409_s_at	EIF1AY	eukaryotic translation initiation factor 1A, Y-linked	down
Cohort 2				
1	239380_at	C5orf27	chromosome 5 open reading frame 27	down
2	203032_s_at	FH	fumarate hydratase	down
3	224588_at	XIST	X inactive specific transcript (non-protein coding)	up
4	238013_at	PLEKHA2	pleckstrin homology domain containing, family A, member 2	down
5	201909_at	RPS4Y1	ribosomal protein S4, Y-linked 1	down
6	209773_s_at	RRM2	ribonucleotide reductase M2	up
7	220116_at	KCNN2	small conductance calcium-activated channel subfamily N member 2	down
8	1555175_at	PBLD	phenazine biosynthesis-like protein domain containing	down
9	227614_at	HKDC1	hexokinase domain containing 1	up
10	204409_s_at	EIF1AY	eukaryotic translation initiation factor 1A, Y-linked	down
Cohort 3				
1	223579_s_at	APOB	apolipoprotein B	up
2	227556_at	NME7	NME/NM23 family member 7	up
3	1558199_at	FN1	fibronectin 1	down
4	227621_at	WTAP	Wilms tumor 1 associated protein	up
5	217878_s_at	CDC27	cell division cycle 27	down
6	229457_at	ANKHD1	ankyrin repeat and KH domain containing 1	down
7	213956_at	CEP350	centrosomal protein 350 kDa	up
8	212291_at	HIPK1	homeodomain interacting protein kinase 1	up
9	1554241_at	COCH	cochlin	up
10	201775_s_at	EFCAB14	EF-hand calcium binding domain 14	down

### Validation of the expression of the core genes in the clinical samples

**Fig. 4:**
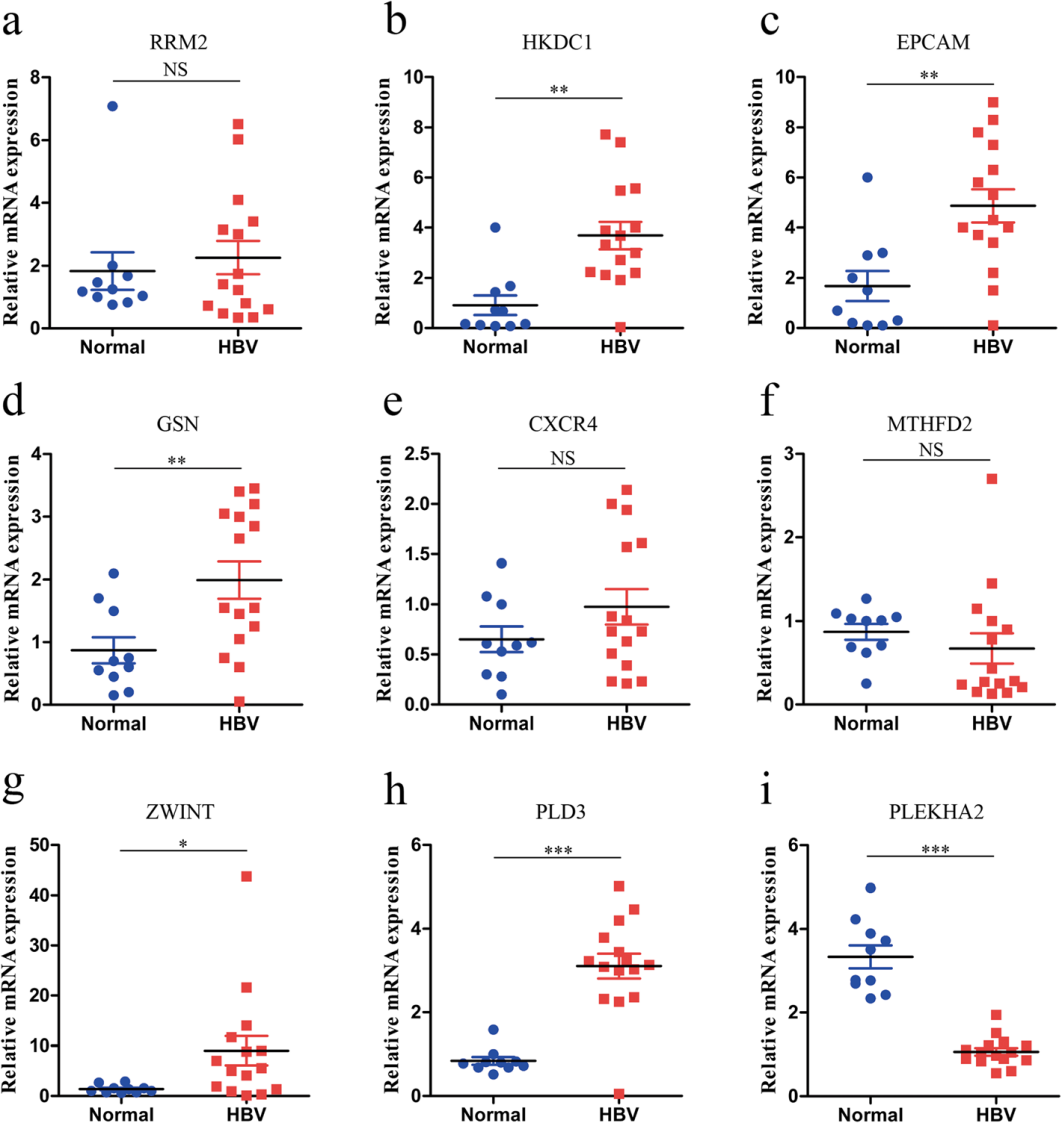
**a**-**i** qRT-PCR validation of the 9 co-expressed DEGs in 15 HBV infected liver and 10 normal liver tissues. *P < 0.05, **P < 0.01, ***P < 0.001, NS represents no significant differences, statistical analyses were performed using non-paired *t* tests

To determine which genes correlated with the HBV infection, we used qRT-PCR to detect the expression of 9 DEGs in 15 liver samples of the liver transplantation patients. Ten liver samples from donors (HBV-negative) were used as controls. We found that the mRNA expression of HKDC1, EPCAM, GSN, ZWINT and PLD3 was significantly increased in the HBV infected liver samples compared to the normal liver samples (Fig. 4b-d, g-h, *P* < 0.05 for all), and the mRNA expression of PLEKHA2 was significantly decreased (Fig. 4i, *P* < 0.001); these findings are consistent with the results of the bioinformatics analysis done above. However, there was no statistically significant difference in the expression of RRM2, CXCR4 and MTHFD2 between the HBV infected and the normal liver samples (Fig. 4a, e–f, *P* > 0.05).

### Biological process analysis

We studied the biological functions of the DEGs by using a GO analysis. The corresponding top 20 biological processes from the 3 cohorts are shown in Table 2. The biological process analysis revealed the following 11 common biological functions in the 3 cohorts, viz., “immune response” and “small molecule metabolic process” “cell adhesion”, “signal transduction”, “negative regulation of apoptotic process”, “apoptotic process”, “mitotic cell cycle”, “protein phosphorylation”, “axon guidance”, “blood coagulation” and “negative regulation of transcription from RNA polymerase II promoter”. The 6 common biological processes in Cohort 1 and Cohort 2 are inflammatory response, negative regulation of transcription (DNA-dependent), protein complex assembly, response to virus, regulation of immune response and dendritic cell chemotaxis. While, there were no common biological process either in Cohort 1 and Cohort 3 or in Cohort 2 and Cohort 3.

**Table 2:** Top 20 biological processes by GO BP analysis in 3 cohorts

Rank	Cohort 1	Cohort 2	Cohort 3
1	immune response	immune response	small molecule metabolic process
2	small molecule metabolic process	small molecule metabolic process	signal transduction
3	inflammatory response	cell adhesion	immune response
4	cell adhesion	inflammatory response	positive regulation of transcription from RNA polymerase II promoter
5	signal transduction	signal transduction	negative regulation of apoptotic process
6	negative regulation of apoptotic process	negative regulation of apoptotic process	transcription, DNA-dependent
7	apoptotic process	negative regulation of transcription, DNA-dependent	innate immune response
8	negative regulation of transcription, DNA-dependent	axon guidance	intracellular protein kinase cascade
9	mitotic cell cycle	protein phosphorylation	virus-host interaction
10	protein phosphorylation	protein complex assembly	apoptotic process
11	protein complex assembly	response to virus	protein phosphorylation
12	response to virus	blood coagulation	blood coagulation
13	axon guidance	response to organic cyclic compound	positive regulation of transcription, DNA-dependent
14	response to organic cyclic compound	mitotic cell cycle	positive regulation of apoptotic process
15	regulation of immune response	regulation of immune response	negative regulation of transcription from RNA polymerase II promoter
16	response to toxic substance	negative regulation of transcription from RNA polymerase II promoter	cell adhesion
17	blood coagulation	chemotaxis	mitotic cell cycle
18	chemotaxis	platelet activation	transmembrane transport
19	negative regulation of transcription from RNA polymerase II promoter	apoptotic process	axon guidance
20	dendritic cell chemotaxis	dendritic cell chemotaxis	positive regulation of cell migration

*GO* gene ontology, *BP* biological process

### Pathway analysis

KEGG pathway analysis was used to investigate the signaling pathway based on the identified DEGs. Table 3 shows the top 20 pathways of the 3 cohort. Among them, PI3K-Akt signaling pathway, focal adhesion, HTLV-I infection, cytokine-cytokine receptor interaction, metabolic pathways, NF-κB signaling pathway, influenza A and chemokine signaling pathway were the 8 common pathways related to HBV infection and response of IFN-α treatment. In addition, extracellular matrix (ECM)-receptor interaction, cell adhesion molecules (CAMs), p53 signaling pathway, viral carcinogenesis, amoebiasis, protein digestion and absorption, proteoglycans in cancer, cell cycle and hepatitis B were the other intersecting pathways in pairwise comparisons.

**Table 3:** Top 20 pathways by KEGG pathway analysis in 3 cohorts

Rank	Cohort 1	Cohort 2	Cohort 3
1	Chemokine signaling pathway	Chemokine signaling pathway	PI3K-Akt signaling pathway
2	Cytokine-cytokine receptor interaction	Cytokine-cytokine receptor interaction	Influenza A
3	Focal adhesion	Focal adhesion	Metabolic pathways
4	PI3K-Akt signaling pathway	PI3K-Akt signaling pathway	Cytokine-cytokine receptor interaction
5	HTLV-I infection	HTLV-I infection	Chemokine signaling pathway
6	Metabolic pathways	Metabolic pathways	HTLV-I infection
7	ECM-receptor interaction	Cell adhesion molecules (CAMs)	Herpes simplex infection
8	Cell adhesion molecules (CAMs)	ECM-receptor interaction	Toxoplasmosis
9	p53 signaling pathway	p53 signaling pathway	Hematopoietic cell lineage
10	Rheumatoid arthritis	Amoebiasis	Protein processing in endoplasmic reticulum
11	Viral carcinogenesis	Proteoglycans in cancer	NF-kappa B signaling pathway
12	Amoebiasis	Viral carcinogenesis	Insulin signaling pathway
13	Protein digestion and absorption	Rheumatoid arthritis	Leukocyte transendothelial migration
14	Proteoglycans in cancer	Influenza A	Focal adhesion
15	NF-kappa B signaling pathway	Protein digestion and absorption	Phagosome
16	Cell cycle	Hepatitis B	Measles
17	Pathways in cancer	NF-kappa B signaling pathway	Adipocytokine signaling pathway
18	Toll-like receptor signaling pathway	Fc gamma R-mediated phagocytosis	Antigen processing and presentation
19	Influenza A	Cell cycle	Adherens junction
20	Hepatitis B	Malaria	Leishmaniasis

*KEGG* Kyoto Encyclopedia of Genes and Genomes

### Gene signal network analysis

**Fig. 5:**
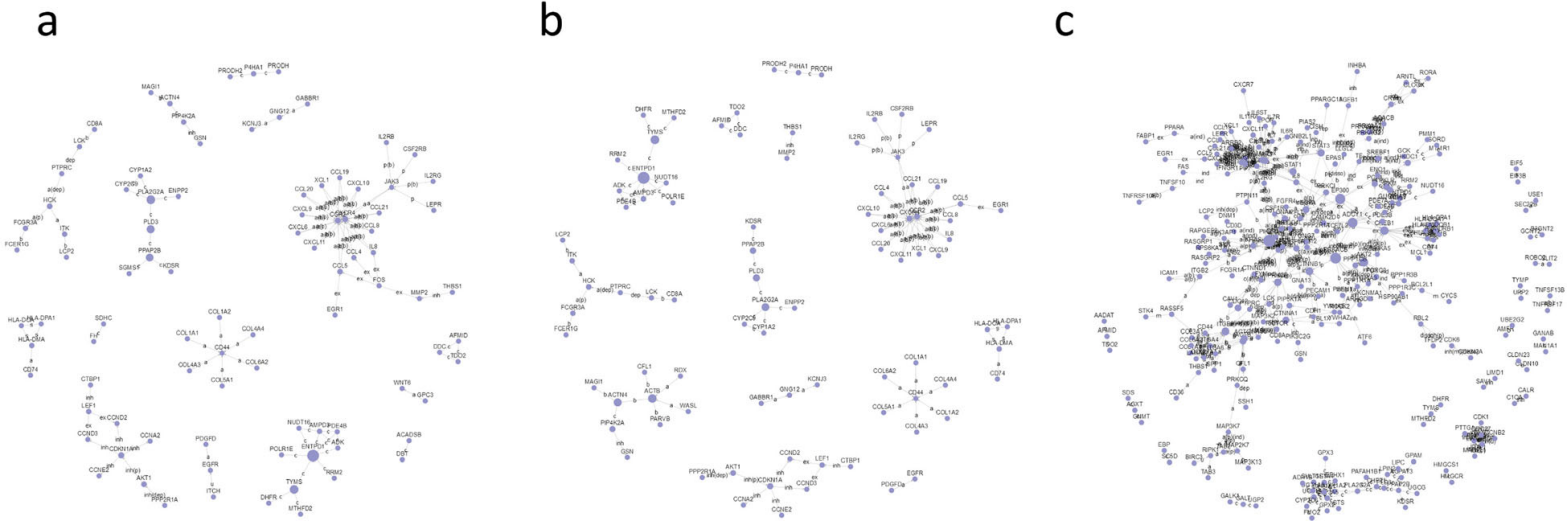
Gene signal network in the 3 cohorts. Genes-genes network derived from proteins-proteins interaction according to GO and KEGG by using GCBI platform. The dots represent hub genes, and the size represents the betweenness centrality value (the greater the value is, the more important the gene is). Arrows represent the relationship between upstream and downstream

On the basis of the interactions between the genes and the genes in KEGG database, the gene signal network analysis was constructed. In the gene signal network analysis, a total of 173, 162, 506 hub nodes (hub genes) in cohort 1, cohort 2 and cohort 3 were identified, respectively (Fig. 5). Table 4 shows the top 10 hub genes involved in the gene signal network in each cohort. Among them, PLA2G2A, CXCR4, GNG12 and CXCL11 were the 4 common genes of the gene signal network (Fig. 7b).

**Table 4:** Top 10 genes in gene signal network in 3 cohorts

Rank	Gene symbol	Gene description	Degree
Cohort 1			
1	ENTPD1	ectonucleoside triphosphate diphosphohydrolase 1	56
2	TYMS	thymidylate synthetase	30
3	PLA2G2A	phospholipase A2, group IIA	28
4	PLD3	phospholipase D family, member 3	24
5	PPAP2B	phosphatidic acid phosphatase type 2B	22
6	CDKN1A	cyclin-dependent kinase inhibitor 1A	8
7	CCR2	chemokine (C-C motif) receptor 2	7
8	CXCR4	chemokine (C-X-C motif) receptor 4	7
9	AKT1	v-akt murine thymoma viral oncogene homolog 1	5
10	CCL5	chemokine (C-C motif) ligand 5	4
Cohort 2			
1	ENTPD1	ectonucleoside triphosphate diphosphohydrolase 1	56
2	TYMS	thymidylate synthetase	30
3	ACTB	actin, beta	28
4	ACTN4	actinin, alpha 4	24
5	PLA2G2A	phospholipase A2, group IIA	22
6	PLD3	phospholipase D family, member 3	16
7	PPAP2B	phosphatidic acid phosphatase type 2B	10
8	CDKN1A	cyclin-dependent kinase inhibitor 1A	8
9	PIP4K2A	phosphatidylinositol-5-phosphate 4-kinase, type II, alpha	7
10	CCR2	chemokine (C-C motif) receptor 2	6.5
Cohort 3			
1	PRKACB	protein kinase, cAMP-dependent, catalytic, beta	18
2	EP300	E1A binding protein p300	18
3	ADCY1	adenylate cyclase 1	18
4	CREB1	cAMP responsive element binding protein 1	17
5	STAT1	signal transducer and activator of transcription 1	15
6	CTNNB1	catenin (cadherin-associated protein), beta 1, 88 kDa	15
7	ITGB5	integrin, beta 5	15
8	PRKCB	protein kinase C, beta	14
9	STAT3	signal transducer and activator of transcription 3	12
10	AKT2	v-akt murine thymoma viral oncogene homolog 2	10

### Gene connections and co-expression networks analyses

**Fig. 6:**
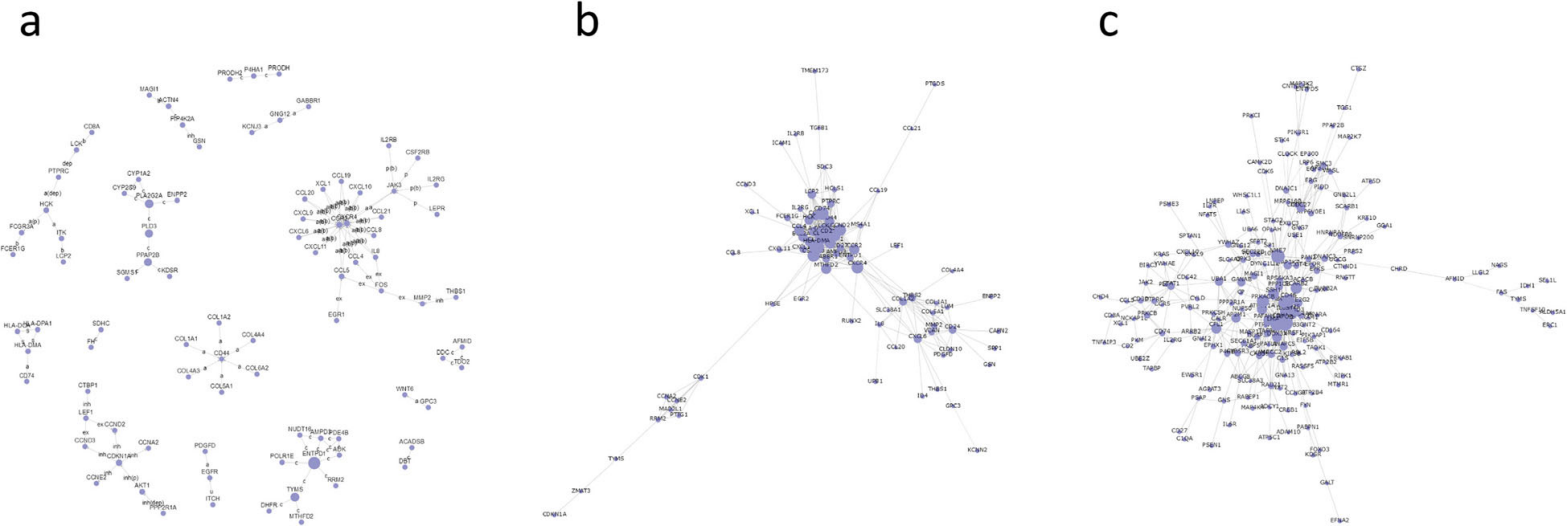
Gene co-expression network of the DEGs in the 3 cohorts. Co-expressed DEGs were integrated into networks using bioinformatics methodology

**Fig. 7:**
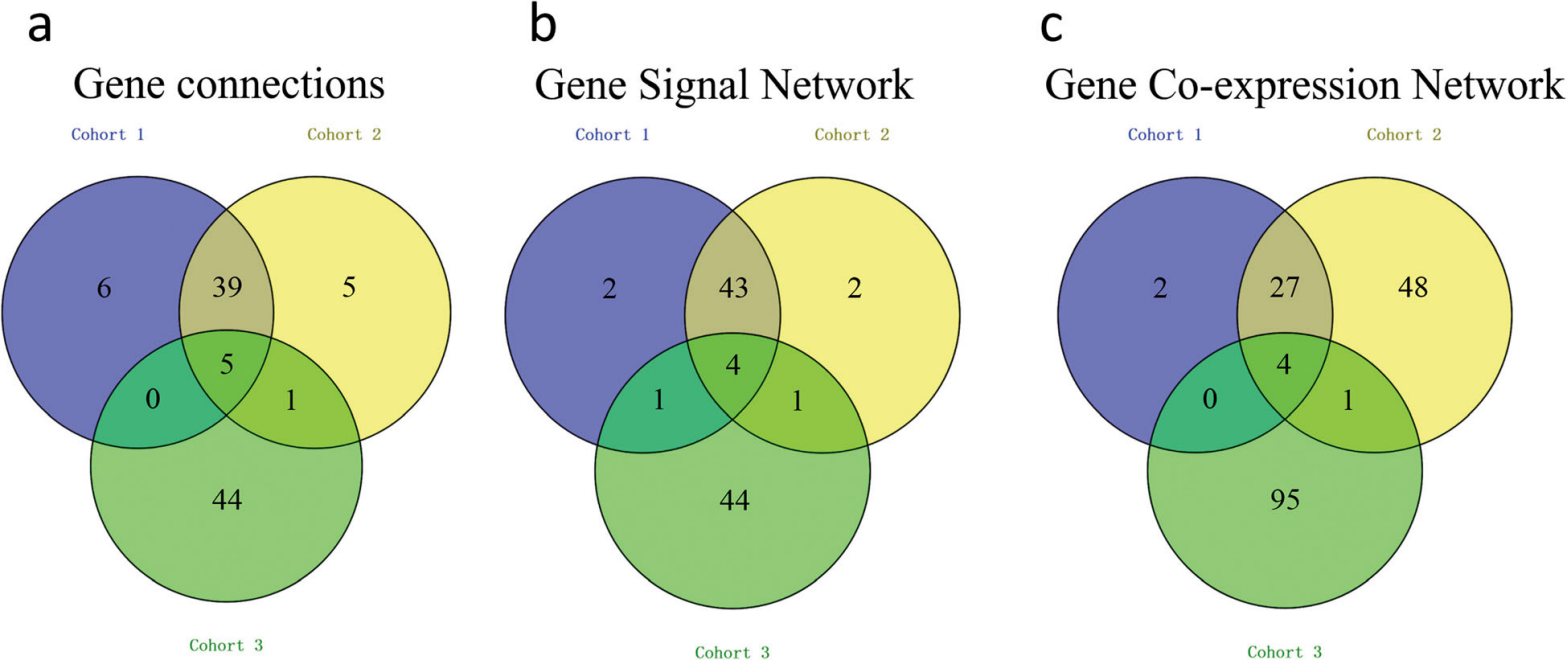
The Venn diagrams show gene connection (**a**), gene signal network (**b**) and the co-expressed genes network (**c**) among the 3 cohorts. **c** The Venn diagram shows that 4 genes have the most gene connections in the gene co-expressed genes network

We picked out 173, 162 and 506 overlapped genes from cohort 1, cohort 2, and cohort 3, respectively and applied them to the gene connections analysis (Fig. 6), which revealed the 5 common genes as ENO1, TDO2, CXADR, MTHFD2 and PSME3 (Fig. 7a). Table 5 shows the top 10 genes involved in the gene co-expression network in the 3 cohorts, amongst which, CD74, CCL5, CXCL10 and PTPRC were the 4 common genes (Fig. 7c). Therefore, CD74, CCL5, CXCL10 and PTPRC were the genes with the most connections in the gene co-expression network.

**Table 5:** Top 10 genes in gene co-expression network in 3 cohorts

Rank	Gene symbol	Gene description	Degree
Cohort 1			
1	CD2	CD2 molecule	17
2	HLA-DMA	major histocompatibility complex, class II, DM alpha	15
3	LCK	lymphocyte-specific protein tyrosine kinase	14
4	CCND2	cyclin D2	12
5	CD8A	CD8a molecule	12
6	ITK	IL2-inducible T-cell kinase	11
7	JAK3	Janus kinase 3	11
8	CSF2RB	colony stimulating factor 2 receptor, beta, low-affinity	10
9	RASGRP1	RAS guanyl releasing protein 1	10
10	CXCR4	chemokine (C-X-C motif) receptor 4	9
Cohort 2			
1	HLA-DMA	major histocompatibility complex, class II, DM alpha	28
2	CD2	CD2 molecule	28
3	CD74	CD74 molecule, major histocompatibility complex, class II invariant chain	26
4	CD8A	CD8a molecule	24
5	CSF2RB	colony stimulating factor 2 receptor, beta, low-affinity	24
6	LCK	lymphocyte-specific protein tyrosine kinase	24
7	CCND2	cyclin D2	23
8	JAK3	Janus kinase 3	23
9	CXCR4	chemokine (C-X-C motif) receptor 4	23
10	RASGRP1	RAS guanyl releasing protein	21
Cohort 3			
1	APOB	apolipoprotein B	26
2	NME7	NME/NM23 family member 7	25
3	LMAN1	lectin, mannose-binding, 1	24
4	CDH1	cadherin 1, type 1, E-cadherin (epithelial)	23
5	CD46	CD46 molecule, complement regulatory protein	23
6	PRKACB	protein kinase, cAMP-dependent, catalytic, beta	22
7	SCARB2	scavenger receptor class B, member 2	20
8	SSH1	slingshot protein phosphatase 1	19
9	PAFAH1B1	platelet-activating factor acetylhydrolase 1b, regulatory subunit 1	18
10	ABCB11	ATP-binding cassette, sub-family B (MDR/TAP), member 11	18

## Discussion

Approximately, 30% of the world’s population shows serological evidence of current or past HBV infection [15]. Chronic HBV infection causes immune-mediated liver damage progressing to cirrhosis and HCC [16]. Thus, early diagnosis and treatment is vital to improve the cure rates of HBV. Many genes could be associated with the susceptibility and development of HBV infection. Previous study reported that the low copy number of TLR7 gene is a risk factor for chronic HBV infection but is not associated with later stages of the disease progression [17]. In the present study, we extracted the gene expression data of HBV infected liver tissues and normal liver tissues from 3 GEO datasets on which we performed comprehensive bioinformatics analyses. Differential expression analyses in GCBI were used to identify the co-expressed DEGs, common biological processes and pathways between HBV infected liver tissues and normal liver tissues, liver biopsy samples of IFN-α responders before and after treatment.

The GCBI comprehensive analysis platform analyses revealed the following genes: RRM2, HKDC1, EPCAM, GSN, CXCR4, MTHFD2, ZWINT, PLD3 were the up-regulated co-expressed DEGs, and PLEKHA2 was the down-regulated co-expressed DEG in 3 different cohorts. This indicates that the 9 genes are potential biomarkers for distinguishing the HBV infection and predicting responders of IFN-α based therapy. To further validation the data in clinical samples, the mRNA expression data showed that the 5 up-regulated DEGs (HKDC1, EPCAM, GSN, ZWINT and PLD3) were significantly increased and PLEKHA2 (the down-regulated DEG) was significantly decreased in HBV infected compared to the normal liver samples. It is known that RRM2 expression is essential for the synthesis of the HBV genomic DNA and cccDNA in the human liver cells, moreover, RRM2-targeting drugs constitute a novel category of candidates for the treatment of HBV-related diseases [18]. However, there were other evidence [19, 20] demonstrated that HBV induces the activation of the RRM2 expression selectively in nonproliferating cells. This may explain why the expression of RRM2 could not be verified in our clinical samples. Previous researches have shown that EPCAM plays a critical role in the process of the HBV-mediated hepatocarcinogenesis [21–23]. CXCR4, one of the chemokine receptors, function as a co-receptor for the T-cell line trophic strains of HIV-1. The expression of CXCR4 in the CD8 memory T cell may be used as biomarkers for predicting the outcomes of the treatment of the chronic HBV patients [24]. Wald’s studies [25] suggest an important role for the CXCL12/CXCR4 pathway in the recruitment and retention of the immune cells in the liver during the chronic HCV and HBV infection. Although some literatures report that CXCR4 is closely related to HBV infection [24, 25], others [26] have shown the opposite effect. In a large case-control study, Jae Youn Cheong et al. reported that CXCR4 gene polymorphisms was not associated with the outcome of the HBV infection [26]. Therefore, the relations between CXCR4 gene and HBV infection remain controversial. It is reported that higher levels of urinary 8-oxo-GSN are more likely to have a high degree of fibrosis in the liver injury patients with HBV infection [27]. Previously, it has been shown that MTHFD2 [28] and ZWINT [29] are important oncogenes which are overexpressed in HCC and contribute to the progression of HCC. However, to our knowledge, there are no researches to show the relationship between MTHFD2 and HBV infection. Thus, the expression and function of MTHFD2 remain to be studied in larger clinical samples and HBV related disease models. These results imply that the abnormal co-expressed genes are involved in the progression of HBV to cirrhosis or liver cancer. Moreover, to validate the reliability of the microarray data, similar results were observed in qRT-PCR analyses in clinical samples in the current study.

From the pathway analysis, we identified the 8 aberrantly expressed signaling pathways in HBV infection. The activation of the PI3K-Akt signaling could regulate replication of the HBV in the liver cells [30], and also play an important role in promoting tumorigenesis of the HBV-associated HCC [31, 32]. Hepatitis B protein X was found to interact with the focal adhesion protein, that blocked the molecular linkage between the actin filament to weaken the intercellular adhesion [33, 34]. Chennari’s study [35] indicated that the epidemiology of HTLV-I and HBV co-infection is related to the endemicity of HBV. In a systematic bioinformatic analysis, Tang et al. [36] found that the human La protein may play an important role in the development and progression of HBV through a cytokine-cytokine receptor interaction and other pathways. In primary rat hepatocytes, Lamontagne et al. [37] showed the metabolic consequences of an HBV infection, as a panel of 7 metabolites which were altered by HBV at different time points. A recent study demonstrated that activation of the NF-κB pathway causes inflammation and liver damage among patients with chronic HBV infection [38]. Changes in the chemokine levels following entecavir [39] or telbivudine [40] treatment are associated with response to antiviral therapy in chronic hepatitis B patients. In the present study, we found that the PI3K-Akt signaling pathway, focal adhesion, HTLV-I infection, cytokine-cytokine receptor interaction, metabolic pathways, NF-κB signaling pathway, influenza A and chemokine signaling pathway were the 8 common pathways related to HBV infection. It is interesting to note that HTLV-1, influenza, measles etc. appear in the list of the top 20 in the 3 cohorts. The prevalence of HTLV-1 and HBV co-infection is high in certain indigenous Australian populations [41]. According to a population-based analysis [42], annual influenza vaccination can reduce the risk of hospitalization and mortality in patients with chronic HBV infection. It implies that the influenza-target therapy may have some effect on HBV infection. In a prospective study in diabetic children [43], the authors found a reduced efficacy of measles vaccination in the anti-HBs (−) patients, compared to the anti-HBs (+) patients. Thus, we can infer that there is an inherent correlation between HBV and measles. In the high endemic areas, HBV and HCV/HBV and HDV co-infection is fairly common [44–46]. Although HCV and HDV infections have an important effect on the gene expression in co-infections with HBV, we didn’t find HCV and HDV co-infection ranking in the top 20 DEGs. This may be explained by population differences and geographical distributions of the clinical samples. The issue still remains about how the co-expressed DEGs influence the pathways or other mechanisms in the progression of HBV infection in humans.

In our gene signal network analysis, we identified PLA2G2A, CXCR4, GNG12 and CXCL11 as the 4 common genes in the 3 cohorts. PLA2G2A is a key enzyme of the arachidonic acid synthases. A previous study [47] demonstrated that the serum levels of PLA2G2A are associated with the progression of HBV-related diseases, and HBV can upregulate the expression of PLA2G2A. Another study [48] found that CXCR4 was closely associated with the development of the HBV-related hepatitis. As compared to the healthy and asymptomatic HBV carriers, expression of CXCL10 and CXCL11 were elevated in patients with chronic active HBV and had positive correlation with ALT levels [49]. ENO1, TDO2, CXADR, MTHFD2 and PSME3 were 5 common genes identified in the gene connections analysis. Xiang Chun D. et al. found that ENO1 expression was upregulated in the HBV-infected liver tissues and cells; moreover, silencing ENO1 resulted in a significant reduction in HBV replication [50]. Additionally, we found that CD74, CCL5, CXCL10 and PTPRC were the 4 common genes in terms of the gene co-expression network. The serum level of CCL5 is a reliable marker that can predict disease progression in chronic hepatitis B patients [51]. The intrahepatic expression of CXCL10 was significantly increased in the HBeAg (+) compared to the HBeAg (−) patients [52]. And the upsurge of the serum HBV load significantly correlated with the increase of CXCL10 [53].

We acknowledge that there were some shortcomings and limitations in our study. First, we focused on the up-regulated and down-regulated genes without analyzing the contra-regulated genes. Second, we could not figure out if the interactions in the protein-protein interaction network were direct or indirect. Additionally, we have not verified the DEGs in the liver specimen of IFN-α responders before/after treatment due to a lack of clinical cases in our center. It needs to be validated in an independent cohort to verify that these genes in the chronic HBV patients underwent IFN-α therapy. Further, though we conducted the validation of DEG expression, we did not validate the gene function or signaling pathways in clinical samples. Further studies considering these aspects are needed in the future.

## Conclusions

To summarize, we used the GCBI bioinformatics analysis platform to study DEGs between HBV infected liver tissues and normal liver tissues, liver biopsy samples of IFN-α responders before and after treatment, which identified that 9 DEGs (8 up-regulated and 1 down-regulated DEGs) and 8 intersecting pathways in the relation network. Then, validation by qRT-PCR in the clinical samples, we concluded that the mRNA expression of 5 up-regulated DEGs was significantly increased and 1 down-regulated DEG was significantly decreased in HBV infected liver. These candidate genes and pathways might become therapeutic targets for HBV infection and further studies are required to elucidate the function and underlining mechanisms of these potential biomarkers in the progression of HBV infection.

## Supplementary information

**Additional file 1: Table S1.** General characteristics of 10 donors and 15 patients who underwent liver transplantation.

**Additional file 2: Table S2.** Sequence of primers used for validation of expression level of co-expressed DEGs.

## Data Availability

The GEO datasets (GSE83148, GSE84044 and GSE66698) analyzed in our study are directly available from GEO database (https://www.ncbi.nlm.nih.gov/gds). Data are also available from the corresponding authors upon reasonable request.

## Abbreviations

CAM: cell adhesion molecule
DEG: differentially expressed gene
ECM: extracellular matrix
EPCAM: epithelial cell adhesion molecule
GCBI: Gene-Cloud of Biotechnology Information
GEO: Gene Expression Omnibus
GO: gene ontology
HBV: hepatitis B virus
HCC: hepatocellular carcinoma
HTLV-I: human T-cell lymphotropic virus type 1
IFN-α: interferon-alpha
KEGG: Kyoto Encyclopedia of Genes and Genomes
NCBI: National Center of Biotechnology Information
qRT-PCR: quantitative real time polymerase chain reaction
SPSS: Statistical Package for the Social Science
WHO: world health organization
