# Supplementary material for: Bioinformatics analysis on multiple Gene Expression Omnibus datasets of the hepatitis B virus infection and its response to the interferon-alpha therapy
Source: BMC Infect Dis. 2020 Jan 29;20:84. doi: 10.1186/s12879-019-4720-x (PMC6990549; doi:10.1186/s12879-019-4720-x)
Supplement: Supplementary file 1 — Additional file 1: Table S1. General characteristics of 10 donors and 15 patients who underwent liver transplantation. [file 12879_2019_4720_MOESM1_ESM.docx]

Table S1. General characteristics of 10 donors and 15 patients who underwent liver transplantation

| Information | Value |
| --- | --- |
| **Donors (n=10)** |  |
| Age-years (mean±SD) | 31.5±11.9 |
| Female (n, %) | 2 (20.0%) |
| BMI-kg/m^2^ (mean±SD) | 25.6±5.2 |
| Cause of death (n, %) |  |
| Trauma | 4 (40.0%) |
| Cerebral vascular accident | 4 (40.0%) |
| Other^a^ | 2 (20.0%) |
| CIT-minutes (mean±SD) | 489.8±157.6 |
| DCD (n, %) | 1 (10.0%) |
| **Patients (n=15)** |  |
| Age-years (mean±SD) | 47.7±8.8 |
| Female (n, %) | 1 (6.7%) |
| BMI-kg/m^2^ (mean±SD) | 24.0±4.5 |
| Child-Pugh classification (n, %) |  |
| A | 0 |
| B | 6 (40.0%) |
| C | 9 (60.0%) |
| Diagnosis (n, %) |  |
| HBV-related decompensated cirrhosis | 6 (40.0%) |
| HBV infection combined with fulminate liver failure | 6 (40.0%) |
| Acute attack of chronic hepatitis | 3 (20.0%) |
| MELD score (mean±SD) | 24.7±8.6 |

SD, standard deviation; BMI, body mass index; CIT, cold ischemia time; DCD, donation after circulatory death; HBV, hepatitis B virus; MELD, model for end‐stage liver disease.

^a^ Includes toxicosis (n=1) and hypoxic brain injury (n=1).
