# Supplementary material for: Bioinformatics analysis on multiple Gene Expression Omnibus datasets of the hepatitis B virus infection and its response to the interferon-alpha therapy
Source: BMC Infect Dis. 2020 Jan 29;20:84. doi: 10.1186/s12879-019-4720-x (PMC6990549; doi:10.1186/s12879-019-4720-x)
Supplement: Supplementary file 2 — Additional file 2: Table S2. Sequence of primers used for validation of expression level of co-expressed DEGs. [file 12879_2019_4720_MOESM2_ESM.docx]

Table S2. Sequence of primers used for validation of expression level of co-expressed DEGs.

| Genes | Sequence (5'-3') |
| --- | --- |
| RRM2 6241-S | GCAAGCGATGGCATAGTAAATGAA |
| RRM2 6241-A | TGGCAATTTGGAAGCCATAGAA |
| HKDC1 80201-S | AGATGACATCCGGACCCGATAC |
| HKDC1 80201-A | GATCAGGATCTGCCGCACAA |
| EPCAM 4072-S | AAGGACACTGAAATAACCTGCTCTG |
| EPCAM 4072-A | TTGATAACGCGTTGTGATCTCC |
| GSN 2934-S | ACGTCATGCTTCTGGACACCTG |
| GSN 2934-A | GAGGCTCAAAGCCTTGCTTCAC |
| CXCR4 7852-S | CCTGCCTGGTATTGTCATCCTG |
| CXCR4 7852-A | ACTGTGGTCTTGAGGGCCTTG |
| MTHFD2 10797-S | CCTCCTTGTTCAGTTGCCTCTT |
| MTHFD2 10797-A | ACTGATCCAAACACATTCGTCCT |
| ZWINT 11130-S | ACGTTTCTGCAGAGGGTAAGG |
| ZWINT 11130-A | CAGCCTTGAAGGACACACCA |
| PLD3 23646-S | AATGGAGATCTGCCTCAATGGAAC |
| PLD3 23646-A | TTCATGACAGCGACGTAGATGAAAC |
| PLEKHA2 59339-S | CTTCATTCTGGACACCCAGGCTA |
| PLEKHA2 59339-A | AGGTCAGCTGCAAAGCTCCAA |

-S, -sense; -A, -anti-sence
